# The effect of music and distraction on pain and anxiety during colonoscopy: a systematic review and meta-analysis

**DOI:** 10.1177/17562848251378236

**Published:** 2025-10-02

**Authors:** Jabed F. Ahmed, Hutan Ashrafian, Ara Darzi, Ferdinando R. Baena, Nisha Patel

**Affiliations:** Endoscopy Unit, Charing Cross Hospital, Imperial College Healthcare NHS Trust, Fulham Palace Road, London W6 8RF, UK; Hamlyn Centre, Imperial College London, London, UK; Hamlyn Centre, Imperial College London, London, UK; Hamlyn Centre, Imperial College London, London, UK; Hamlyn Centre, Imperial College London, London, UK; Endoscopy Unit, Charing Cross Hospital, Imperial College Healthcare NHS Trust, London, UK; Hamlyn Centre, Imperial College London, London, UK

**Keywords:** anxiety, colonoscopy, distraction, music, pain

## Abstract

**Background:**

Music has been shown to reduce pain and anxiety in patients undergoing colonoscopy. Distraction, a newer technique with less available evidence, has shown similar effective outcomes.

**Objectives:**

This systematic review and meta-analysis evaluate the current evidence available on music and task distraction and its potential to reduce pain in colonoscopy.

**Design:**

The study was performed within PRISMA guidelines and registered with PROSPERO. Inclusion criteria comprised peer-reviewed randomised controlled trial publications in English. Exclusion criteria comprised duplicate studies, non-peer-reviewed and non-English studies.

**Methods:**

A literature search was conducted with Medline, Embase, Cochrane and Google. Two independent clinicians reviewed the studies to avoid inclusion bias. Visual analogue score mean pain and Spielberger State-Trait Anxiety Inventory (STAI) mean anxiety were collected. Inverse variance DerSimonian-led meta-analytical approach was conducted using a random effects model and statistical software STATA.

**Results:**

Music intervention reported a significant (*p* < 0.05) weighted mean reduction of 1.50 for pain scores (95% CI 0.69–2.31) and a significant weighted mean reduction of 3.56 for anxiety scores (95% CI 0.86–6.27).

Distraction intervention reported a significant weighted mean reduction of 1.59 for pain scores (95% CI 0.79–2.39) and a significant weighted mean reduction of 7.49 for anxiety scores (95% CI 3.64–11.35). There was high heterogeneity recorded for both pain and anxiety studies (*I*² >90%).

**Conclusion:**

Music and distraction intervention has the ability to be introduced at minimal cost. Furthermore, no changes to endoscopy infrastructure are required. This allows a clinical real-world option that is immediately implementable for patients. This meta-analysis has demonstrated that there is a potential role for music and task distraction to reduce pain and anxiety for patients undergoing a colonoscopy. It supports a low cost and safe option for patients who may not be eligible for sedation. Whilst the body of evidence is growing, it is plausible to claim these interventions can be implemented and established into daily clinical practice.

## Plain language summary

The effect of music and distraction on pain and anxiety during colonoscopy; a review and analysis of the literature

Music and distraction have been shown to reduce pain and anxiety for patients undertaking a colonoscopy.

This review and analysis evaluates the current evidence available in literature.

Studies were evaluated using established criteria and the two factors looked at where pain scores and anxiety scores reported by patients. Scores were compared between no intervention and either the music or distraction technique.

The results were that both music and distraction reduced both pain and anxiety scores for patients. The results were also of statistical significance adding further weighting that this is a direct impact of these interventions.

There is a possibility to introduce these interventions at minimal cost with no major changes needed to an endoscopy department and therefore could be implemented in a short time frame also.

In summary this review and analysis has highlighted that there is a role for music and task distraction to reduce pain and anxiety for patients undertaking a colonoscopy. It suggests a safe and low-expense option for patients who may not be able to have sedation.

## Introduction

The diagnostic and therapeutic role of colonoscopy in the investigation of bowel symptoms has been widely documented. It is the gold standard procedure for detecting colorectal cancer.^1,2^ A significant barrier to patients attending for colonoscopy is the anticipated pain and discomfort they may experience, which can be up to 84%.^3^ This can occur for both first-time and reattending patients.^4^ Anxiety has also been demonstrated to be an important predictor of patient co-operation during a colonoscopy procedure.^5^ Pain and anxiety can therefore result in reduced engagement with clinical services, which can have a detrimental effect on precancerous and cancer detection rates.^6^

A frequently utilised method to reduce the level of anticipated discomfort and anxiety is to use medication administered prior to the commencement of a colonoscopy procedure. Pain relief and sedation administered include Fentanyl and Midazolam. This may not be a viable option in specific scenarios, such as attendance without an escort, common for patients who live alone. A medical history of renal or liver failure, advanced age, drowsiness and alcohol abuse are other possible contraindications.^7^ The required post-procedure care for patients who are administered sedation can require prolonged observation, resuscitation support such as intravenous fluids and in some cases reversal of the medication administered. Entonox (Nitrous oxide and air) is an alternative option to reduce pain and anxiety, but also has contraindications to its use, such as chronic lung conditions, recent ear and eye procedures and recent head injury.^8^ Therefore, there is a possibility of a significant proportion of patients who are susceptible to a higher likelihood of pain and discomfort during colonoscopy due to no viable option to improve their pain and anxiety. It highlights a need to improve or modify current methods to mitigate anticipated pain, discomfort and anxiety peri-procedure in the most effective manner possible.

The advantages of music in medical settings have been previously documented, such as in surgical operating theatres.^4,9–11^ A wide array of musical genres has been implemented in past studies with the most evidence for classical music reported.^12^ This is due to a slow tempo of 60–80 beats per minute of sound and no significant rhythm, allowing relaxation, sedation and a decreased heart rate.^4,13,14^

Task distraction techniques as an alternative to music have been implemented historically, but have a less developed evidence base. Methods are relatively inexpensive and non-invasive, such as smartphone distraction, with a high likelihood of patients possessing a compatible device, allowing easy accessibility.^15^ The benefits have been demonstrated in burn units where virtual reality (VR) has been used for pain management.^16^ Distraction provides a positive focus and attention on a more pleasant sensation compared to what may be being experienced.^15^

Task distraction can consist of video observation, VR observation and manual tasks. Multiple modalities can also be used consecutively, such as audio with video playback.^17–19^

VR and multimodal distraction engage specific mechanistic and neurophysiological pathways. These include attention modulation, in which attention can be redirected away from painful stimuli, thereby reducing pain perception. Pain processing pathways are activated and can reduce the emotional experience of pain and neurochemical pathways can increase serotonin and endogenous opioid neurotransmitters, contributing to analgesia.^20^

Previous meta-analyses have been undertaken looking at the effect of music on colonoscopy (Table 1). However, these reviews have lacked scope, limited analysis and inclusion criteria, which this systematic review and meta-analysis aims to address. This systematic review and meta-analysis will aim to identify the effect of music and distraction on pain and anxiety for patients undergoing a colonoscopy.

**Table 1.** Summary of music and distraction meta-analyses previously undertaken.

Type of meta-analyses	Title	Year	No. of RCTs (no. of patients)	Summarised result
Music	Rudin et al.^21^	2007	6 (641)	Significantly lower anxiety levels with music
	Tam et al.^22^	2008	8 (722)	Significant reduction in the use of sedation with music
	Bechtold et al.^5^	2009	8 (712)	Patients’ overall experience was significantly improved with music. No significant differences were noted for patients’ pain scores
	Wang et al.^4^	2014	21 (2134)	Significantly improved pain score and anxiety score with music
	Heath et al.^23^	2019	11 (988)	Significant reduction in procedure times, patient experience and pain with music
	Sorkpor et al.^24^	2021	7 (622)	Demonstrated a small treatment effect, which was clinically not statistically significant
Distraction	Zhang et al.^25^	2023	5 (301)	Visual only. Significant reduction in pain, no significant change in anxiety or procedure time
	Saab et al.^26^	2024	13 (1439)	Visual and audio-visual. Significant reduction in pain and anxiety

RCT, randomised controlled trials.

Two systematic reviews have been undertaken for distraction, focusing on visual distraction (Table 1). This is the first systematic review and meta-analysis to date reporting on multiple distraction techniques, including smartphone and VR. In addition, subgroup analysis undertaken on distraction reports upon how blinding, previous colonoscopy, sedation, intra-procedural therapy and type of distraction technique impacts pain and anxiety scores.

## Methods

This review was conducted and reported in accordance with the Preferred Reporting Items for Systematic Reviews and Meta-Analyses (PRISMA) statement^27^ (Supplemental Appendix 1). The study protocol was pre-registered with PROSPERO (Registration No: CRD42025639821).

### Search strategy

**Figure 1.**
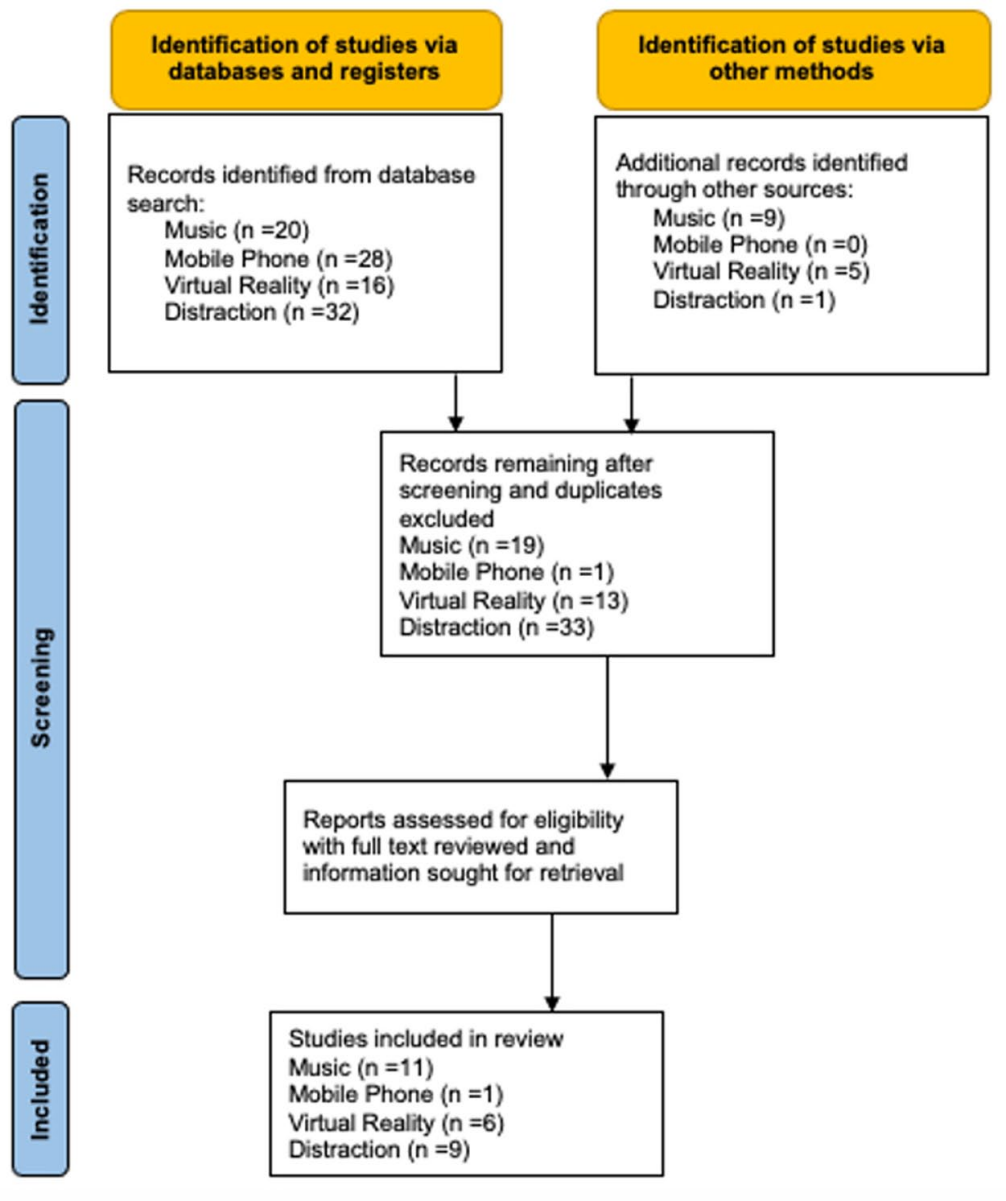
Preferred Reporting Items for Systematic Reviews and Meta-Analyses (PRISMA) flow diagram of study selection. Music, mobile phone, virtual reality and distraction.

A comprehensive literature search was conducted using Medline, Embase, Cochrane Library and Google Scholar databases up to December 2024 (Figure 1). Following this, a manual search was conducted for any additional relevant articles published alongside a review of previous meta-analyses to identify additional relevant published articles.

The full search terms used are described in Supplemental Appendix 1 and include MeSH terms ‘music’; ‘smartphone’; ‘distraction’; ‘virtual reality’ and ‘colonoscopy’.

For studies with full text not available for review, authors were contacted, with no response from two studies, and hence excluded.^28,29^ Major gastrointestinal meeting abstracts were not searched as part of the literature review and the meta-analysis search was limited to randomised controlled trials (RCT).

### Primary and secondary outcomes

The primary outcomes were mean pain and anxiety scores reported by patients undertaking colonoscopy with and without intervention. The mean score was calculated using a graded score scale completed by the patients. The pain score used was the visual analogue score (VAS) developed by Price et al.^30^ and anxiety score used was the Spielberger State-Trait Anxiety Inventory (STAI) developed by Charles Spielberger (1983).^31^

### Inclusion/exclusion criteria

Inclusion criteria were as follows: (1) Patients aged 18 years and older; (2) RCT study; (3) published in English; (4) relevant to the outcomes and (5) a colonoscopy procedure.

Exclusion criteria were as follows: (1) Duplicate publications or no original data present; (2) no full text available; (3) studies not in English; (4) Studies not relevant to the question and (5) a gastroscopy or sigmoidoscopy procedure only.

### Study selection

Two authors (J.A. and N.P.) reviewed the studies with abstracts independently screened for relevance to the questions and outcomes. Duplicate studies and those meeting exclusion criteria were excluded. Full-text review then followed. For any data not identified within the manuscript, the corresponding authors were contacted for additional information or clarification.

### Data extraction

Spreadsheet software was used to collect data from each study. Data extracted consisted of the following: (1) study details of authors, year of publication and journal; (2) study design and if an RCT; (3) mean pain score or mean anxiety score (scale used, range, standard deviation or interquartile range provided).

Three of the music studies and four of the distraction studies required the use of simple mathematical calculations for data extraction. This was to allow statistical comparison to be performed more efficiently. Data was also gathered on how music or distraction was delivered, timing and duration of interventions, previous procedures and the use of sedation, therapy, randomisation and blinding.

### Statistical analysis

An inverse variance DerSimonian-led meta-analytical approach was conducted. Both weighted mean difference and proportional change were calculated. Summary scores and confidence intervals (CIs) were calculated. Significance was set at a *p*-value of <0.05. Meta-analysis of data was conducted using a random effects model. Where available, missing data were imputed, such as standard deviation, during statistical analysis.

Inter-study heterogeneity was assessed using the *I*^2^ value to measure the degree of variation not attributable to chance alone. A value of 0–29 was considered low heterogeneity, 30–59 was considered medium heterogeneity, and anything 60 or above was considered high heterogeneity. A sensitivity analysis was also undertaken.

The study was performed in line with Cochrane recommendations and PRISMA guidelines and using the statistical software StataCorp LLC (version 15).

### Study quality assessment

Study quality was independently assessed using the Jadad scale for RCTs. Studies with a Jadad scale score of 3 or more were considered good studies. All studies were included in the statistical analysis and discussion (Supplemental Appendix 2a/2b). Publication bias analysis was also undertaken.

## Results

### Eligible studies

Music: A total of 29 music studies were identified from database searches (Figure 1). Classical music was the most popular genre, followed by the traditional music of the originating study country. After removal of duplicates, 19 studies for music were screened and identified for full-text review. After further evaluation and application of the exclusion criteria, 11 studies were eligible for inclusion in this meta-analysis (Table 2).^15,16,33–40^

**Table 2.** Summary of study characteristics from music RCTs.

Study	Type of music	How music played	Blinding	Duration	Use of sedation	No of patients (control/intervention)	Findings (intervention vs control)
Lee et al. 2002^36^	Classical, jazz, popular (Chinese or English language), and Chinese opera	Headphones	Yes	Not specified	Group 2: PCA only Group 3: Music only group could request sedation	110 (55/55)	Pain: non-significant reduction
Binek et al. 2003^34^	Multiple styles of music (non-classical/ light or classical)	Speaker	No	Not specified	Used as per patient request	301 (150/151)	Pain: significant reduction
Bechtold et al. 2006^37^	Album titled “Watermark” by Enya	Speaker	Yes	Not specified	All sedated	166 (81/85)	Pain and anxiety: non-significant reduction
Ovayolu et al. 2006^33^	Turkish	Speaker	No	Before and during procedure	Sedation used. Number of patients not specified	60 (30/30)	Pain and anxiety: significant reduction
Costa et al. 2010^39^	Blues, swing, classic, country, jazz, glam-rock, 50–60–70s rock, instrumental, new age, Celtic, reggae, relaxing, Spanish music, classic Italian pop, modern Italian pop, classic American pop, motion picture sound, native American	Headphones	Yes	Not specified	Used as per patient request. Midazolam and Pethidine.	109 (53/56)	Pain: significant reduction
Martindale et al. 2014^40^	Johann Sebastian Bach (Classical)	Headphones	No	During procedure	Sedation used. Number of patients not specified	34 (17/17)	Pain and anxiety: non-significant reduction
De Silva et al. 2016^16^	Sinhala, Hindi, Classic, and Hip-hop genres	Head-mounted set	Yes	During procedure	All sedated. Midazolam and Pethidine	133 (67/66)	Pain: significant reduction
Ko et al. 2017^35^	Classical music or light music	Speaker (from Ipad)	No	During procedure	No sedation	138 (57/81)	Anxiety: significant reduction
Celebi et al. 2020^32^	Turkish classical	Headphones	No	During procedure	All sedated. Midazolam 2 mg	112 (56/56)	Pain and anxiety: significant reduction
Brix et al. 2022^38^	Instrumental acoustic music with integrated sounds of nature, intended to form a connection with everyone, no matter their taste in music, listening habits and preferences	Headphones 30 min before and then music pillow during procedure	No	Before and during procedure	Used as per patient request.	337 (168/169)	Pain and anxiety: non-significant reduction
Cakir et al. 2023^15^	Acemasiran-type classical Turkish music	Headphones	No	Before and during procedure	No sedation	60 (30/30)	Pain: significant reduction Anxiety: non-significant reduction

RCT, randomised controlled trials.

Task distraction: A total of 82 distraction studies were identified from database searches (Figure 1). After removal of duplicates, 47 studies for distraction were screened and identified for full-text review. After further evaluation and application of the exclusion criteria, 16 studies were eligible for inclusion in this meta-analysis (Table 3).^15,17–20,41–47^

**Table 3.** Summary of study characteristics from distraction RCTs.

Study	Type of distraction	Method of delivery	blinding	No of patients (control/intervention)	Use of sedation	Findings (intervention vs control)
De Silva et al. 2016^15^	Visual	Movie viewed on screen	Yes	134 (67/67)	All sedated. Midazolam and Pethidine	Pain: significant reduction Anxiety: non-significant reduction
Han et al. 2021^41^	Smartphone	Not specified	No	360 (180/180)	none	Pain: significant reduction
Cakir et al. 2021^42^	Virtual reality	Headset	No	60 (30/30)	None	Pain: significant reduction Anxiety: non-significant reduction
Liu et al. 2022^43^	Virtual reality	Headset	Yes	120 (60/60)	None	Pain: significant reduction
Cakir et al. 2023^15^	Stress ball	–	No	60 (30/30)	None	Pain: significant reduction Anxiety: non-significant reduction
Cakir et al. 2023^15^	Virtual reality	Headset	No	60 (30/30)	None	Pain: significant reduction Anxiety: non-significant reduction
Umezawa et al. 2015^44^	Video	Head-mounted display	Yes	60 (30/30)	None	Pain and anxiety: non-significant reduction
Xiaolian et al. 2015^17^	Visual	Soundless DVD with earphones	No	120 (60/60)	None	Pain: significant reduction Anxiety: non-significant reduction
Xiaolian et al. 2015^17^	Audio-visual	DVD with earphones	No	120 (60/60)	None	Pain: significant reduction Anxiety: non-significant reduction
Lee et al. 2004^18^	Visual	Headset	No	105 (53/52)	All sedated. Propofol and Alfentanil	Pain: significant reduction
Lee et al. 2004^18^	Audio-visual	Headset	No	105 (53/52)	All sedated. Propofol and Alfentanil	Pain: significant reduction
Sheng et al. 2020^19^	Video	Headset	No	120 (60/60)	None	Pain and anxiety: significant reduction
Sheng et al. 2020^19^	Audio-visual	Headset	No	120 (60/60)	None	Pain and anxiety: significant reduction
Shamali et al. 2024^45^	Virtual reality	Headset	No	47 (24/23)	Midazolam and Fentanyl 30 patients from both groups	Pain: significant reduction Anxiety: non-significant reduction
Veldhuijzen et al. 2020^46^	Virtual reality	Headset	No	19 (9/10)	Midazolam and alfentanyl	Pain and anxiety: non-significant reduction
Yilmaz et al. 2021^47^	Virtual reality	Headset	No	44 (22/22)	None	Pain and anxiety: significant reduction

RCT, randomised controlled trials.

### Music

A cumulative total of 1590 patients were recruited over 11 music RCT studies. Six studies analysed both pain and anxiety scores, four studies reported on pain scores only and one study on anxiety scores only. Seven studies scored at least three on the Jadad scale for reporting RCTs.^48^ In 4 of the 11 studies, the endoscopist was blinded. Out of these four studies, two reported that pain scores significantly reduced with intervention. No blinded study showed anxiety scores significantly reduced with intervention. Table 2 summarises the RCT studies with music intervention.

#### Pain

Six of the 10 music RCT studies (775 patients) found the intervention group with music to have a significant improvement in pain scores. Four studies reported an improvement but no significant difference.

**Figure 2.**
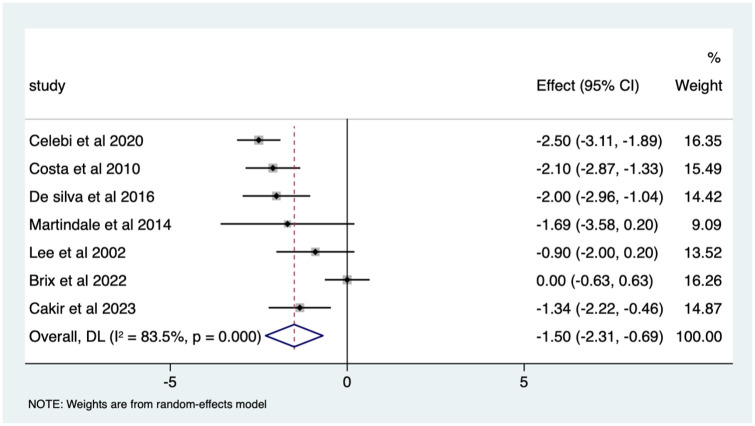
Music RCT pain score Forrest plot. RCT, randomised controlled trials.

Overall, there was a significant (*p* < 0.0005) weighted mean reduction of 1.50 for pain scores with music intervention, with a 95% CI (0.69–2.31; Figure 2).

Overall, there was a significant pain score change of 37% in VAS (*p* < 0.0005) with a 95% CI (0.17–0.57; Supplemental Appendix 3).

The heterogeneity is high with an *I*^2^ value of 97.8%. The average quality score was 3, representing a good score.

#### Anxiety

Three of the seven studies (334 patients) found the intervention group with music to have a significant improvement in anxiety scores. Four studies found an improvement but no significant difference.

**Figure 3.**
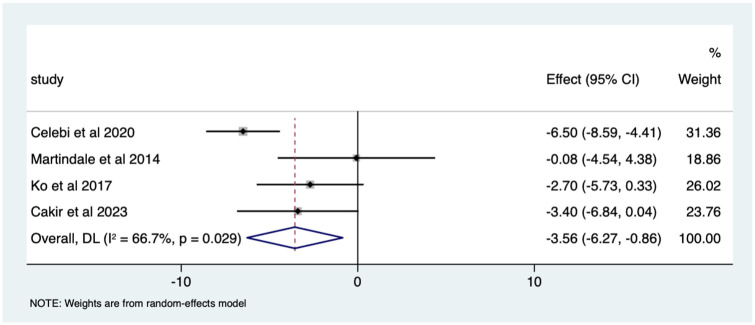
Music RCT anxiety score Forrest plot. RCT, randomised controlled trials.

Overall, there was a significant (*p* < 0.05) weighted mean reduction of 3.56 for anxiety scores with music intervention, with a 95% CI (0.86–6.27; Figure 3).

Overall, there was a significant anxiety score change of 46% in VAS (*p* < 0.0005) with a 95% CI (0.12–0.80; Supplemental Appendix 4). The heterogeneity was high with an *I*^2^ value of 99.2%. The average quality score was 3 representing a good score.

The choice of music varied, with the majority of studies aiming to implement music with an underlying relaxing tone. Six (54.5%) studies had patients listening via head/earphones. Three of these six studies showed significant pain reduction with headphones. One out of four studies had a significant anxiety score reduction with headphones.^32^

Four (30.8%) studies used a speaker. Two out of three^33,34^ reported a significant pain difference and two out of three^33,35^ reported a significant anxiety score difference.

One (7.7%) study used a mounted headset, with significant pain reduction reported.^16^

The onset of music being played was most often during the colonoscopy procedure, with three studies not explicitly describing the start, stop and duration of the music^34,36,37^ and three studies describing starting the music before the procedure.^15,33,38^ Two out of three had a statistically significant decrease in pain and one out of three a statistically significant decrease in anxiety in those studies starting music beforehand.^33,38^

Nine of the 11 music RCT studies used sedation alongside the music intervention, with some studies not detailing the specifics of name, dose, exact quantities used and if administered on patient demand. In the studies that reported sedation; midazolam and pethidine was used. Six studies reported a significant pain reduction and four no significant pain reduction. Five studies measured anxiety and sedation use, with only one study reporting a significant reduction. Two studies measured anxiety with no sedation use, with one study reporting a significant anxiety reduction.

### Task distraction

A cumulative total of 1163 patients were recruited over 12 distraction studies. Nine studies analysed both pain and anxiety scores and three studies reported on pain scores only. Twelve RCT studies assessed pain with 16 distraction techniques used as intervention arms. Nine RCT studies assessed anxiety with 12 distraction techniques used as intervention arms. All 12 studies scored at least 3 on the Jadad scale for reporting RCTs.^48^ In 3 of the 12 studies the endoscopist was blinded and of these three blinded studies, two demonstrated a significant reduction in pain score. No blinded study reported that anxiety scores were significantly reduced. Table 3 summarises the RCTs with distraction intervention.

#### Pain

Fourteen of the 16 techniques found the distraction intervention group to have a significant improvement in pain scale reporting. The remaining two techniques found non-significant improvement.

**Figure 4.**
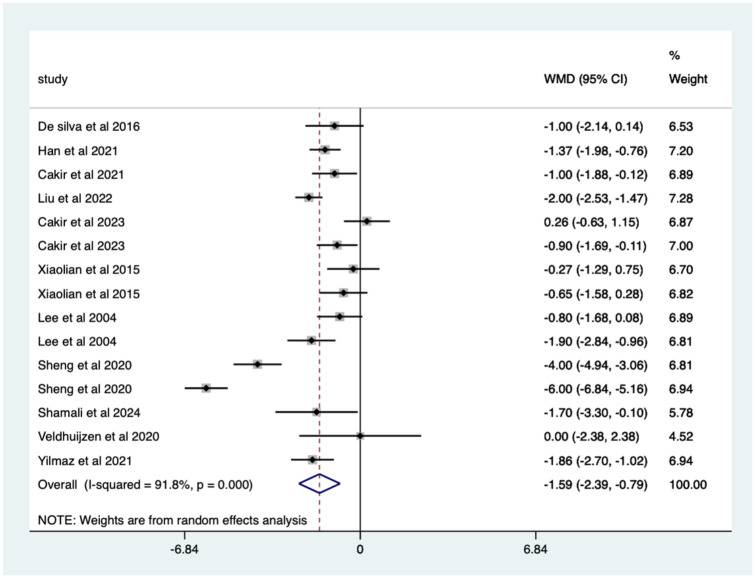
Distraction RCT pain score Forrest plot. RCT, randomised controlled trials.

Overall, there was a significant (*p* < 0.0005) weighted mean reduction of 1.59 for pain scores with distraction intervention, with a 95% CI (0.79–2.39; Figure 4).

The heterogeneity was high with an *I*^2^ value of 91.8%. The average quality score was 3, representing a good score.

#### Anxiety

Three of the nine techniques found the intervention group with distraction to have a significant improvement on the anxiety scale scoring. Six techniques found no significant difference.

**Figure 5.**
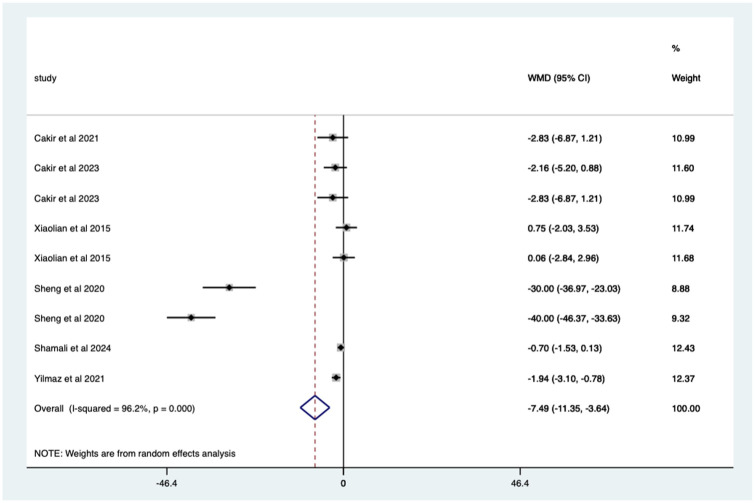
Distraction RCT anxiety score Forrest plot. RCT, randomised controlled trials.

Overall, there was a significant (*p* < 0.0005) weighted mean reduction of 7.49 for anxiety scores with distraction intervention, with a 95% CI (3.64–11.35; Figure 5).

The heterogeneity is high with an *I*^2^ value of 96.2%. The average quality score was 3, representing a good score.

The distraction methods used included five visual/video, four combined audio and video, six VR, one smartphone and one stress ball.

Four out of five visual technique studies found distraction intervention to reduce pain scores significantly. One out of three visual technique studies found that distraction intervention reduced anxiety scores significantly.

All four audio-visual technique studies reported a statistically significant decrease in pain with intervention. One out of three audio-visual technique studies reported a statistically significant decrease in anxiety.

Five out of six VR technique studies reported a significant pain score reduction with intervention, and one VR technique study reported a significant anxiety score reduction.

The smartphone technique study reported a significant reduction in pain with intervention and the stress ball technique study reported a significant pain score reduction and no significant anxiety score reduction.

Four of the 12 studies used sedation alongside the distraction intervention. Three studies reported a significant pain reduction for the five distraction techniques used.

One study used propofol and alfentanyl, whilst three used midazolam and pethidine.

The three studies measuring anxiety and using sedation all reported a non-significant anxiety score reduction.

### Distraction sub-analysis

The 12 distraction studies were analysed in further detail, looking at multiple components which may influence the effect of the intervention. These included:

- Blinding
- Patients who have undertaken a previous colonoscopy
- Sedation
- Undergoing therapeutic intervention
- Distraction type

(All references to significance in the results below refer to a *p*-value of <0.01.)

### Blinding

#### Pain

**Figure 6.**
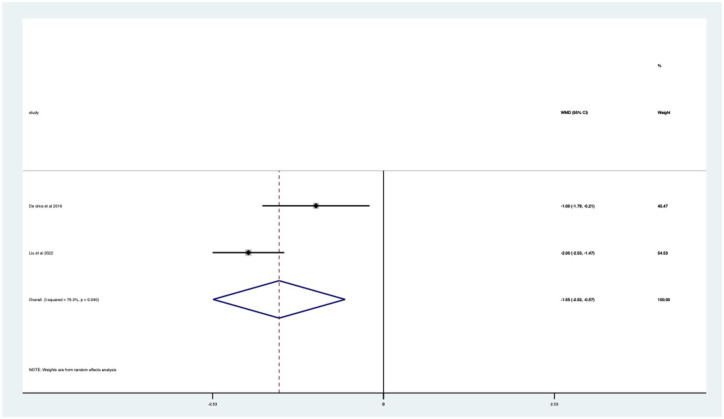
Studies with blinding; pain score Forrest plot.

Studies with blinding – significant mean 1.55 (CI 0.57–2.52) pain score reduction (Figure 6).

**Figure 7.**
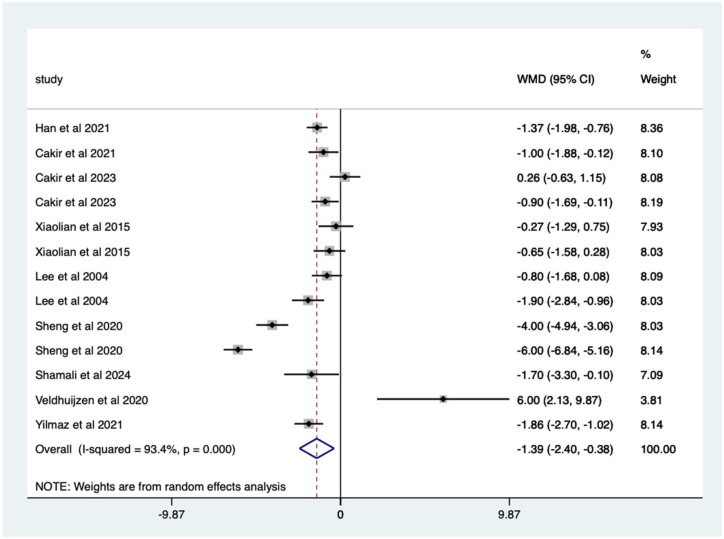
Studies with no blinding; pain score Forrest plot.

Studies with no blinding – significant mean 1.39 (CI 0.38–2.40) pain score reduction (Figure 7).

#### Anxiety

**Figure 8.**
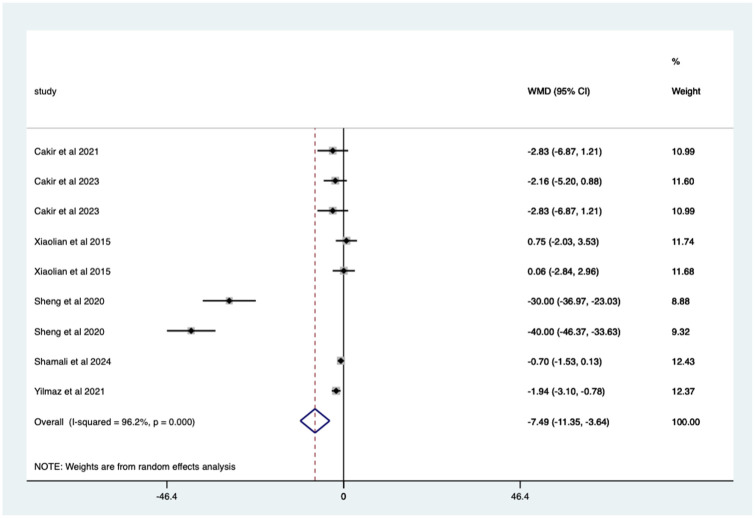
Studies with no blinding; anxiety score Forrest plot.

Studies with no blinding – significant mean 7.49 (CI 3.64–11.35) anxiety score reduction (Figure 8).

Studies with blinding – One distraction study underwent blinding but had incomplete information with no standard deviation data.

### Previous colonoscopy

#### Pain

**Figure 9.**
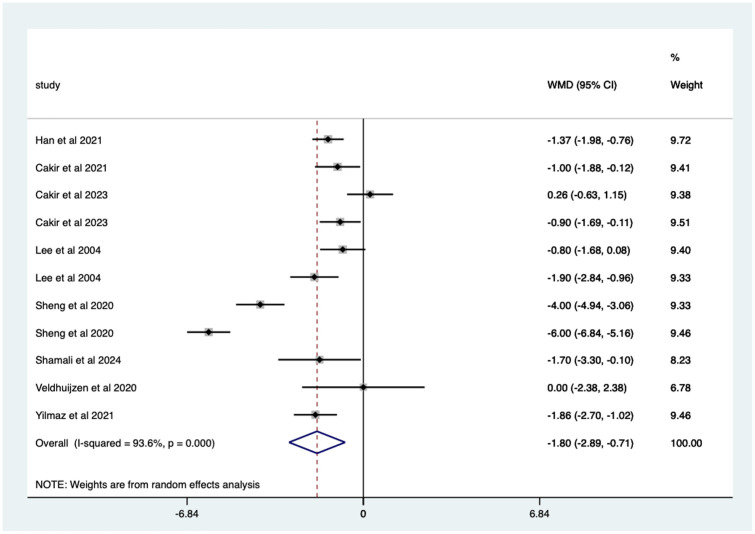
Studies with patients undergoing previous endoscopy; pain score Forrest plot.

Studies with previous colonoscopy – significant mean 1.80 (CI 0.71–2.89) pain score reduction (Figure 9).

**Figure 10.**
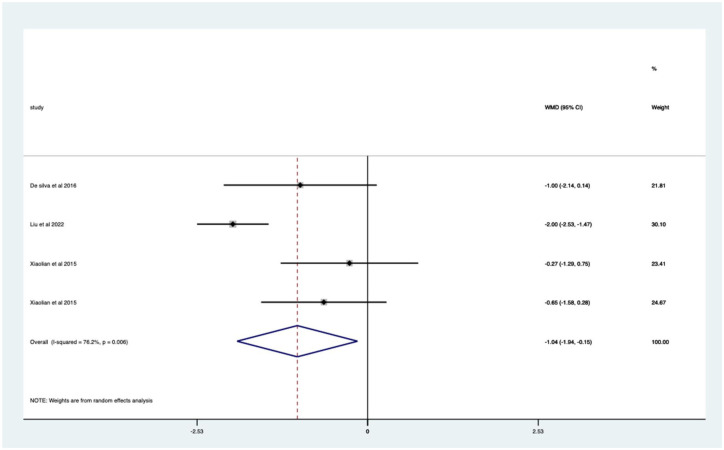
Studies with patients undergoing no previous endoscopy; pain score Forrest plot.

Studies with no previous colonoscopy – significant mean 1.04 (CI 0.15–1.94) pain score reduction (Figure 10).

#### Anxiety

**Figure 11.**
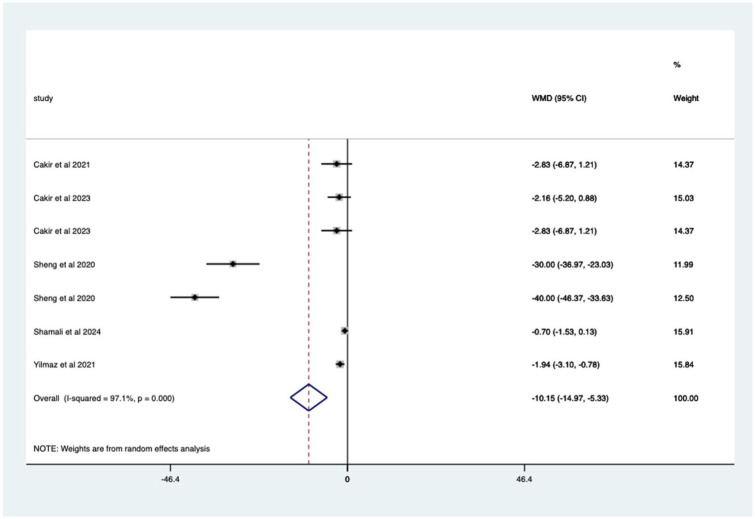
Studies with patients undergoing previous endoscopy; anxiety score Forrest plot.

Studies with previous colonoscopy – significant mean 10.15 (CI 5.33–14.97) anxiety score reduction (Figure 11).

**Figure 12.**
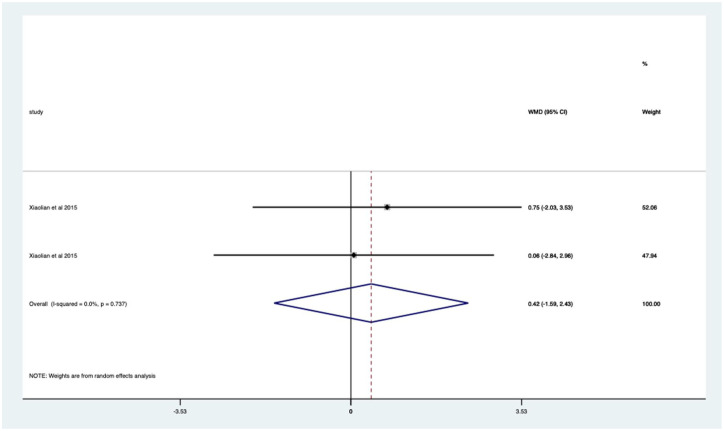
Studies with patients undergoing no previous endoscopy; anxiety score Forrest plot.

Studies with no previous colonoscopy – non-significant mean 0.42 (CI −2.43 to 1.59) anxiety score reduction (Figure 12).

### Sedation

#### Pain

**Figure 13.**
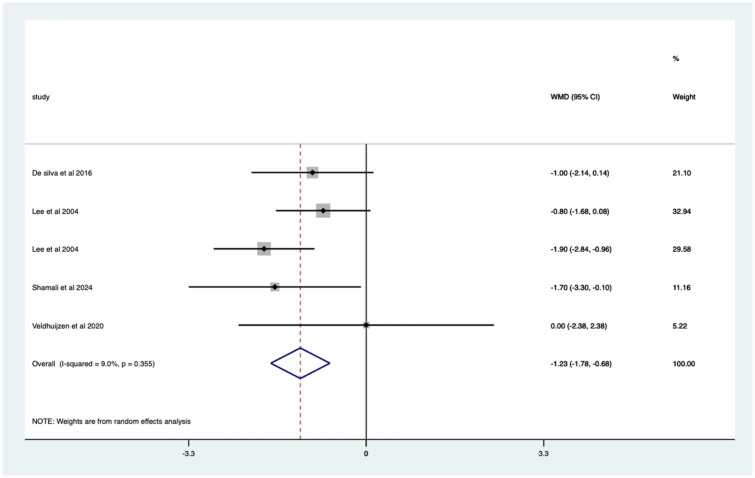
Studies with patients undergoing sedation; pain score Forrest plot.

Studies with sedation – significant mean 1.23 (CI 0.68–1.78) pain score reduction (Figure 13).

**Figure 14.**
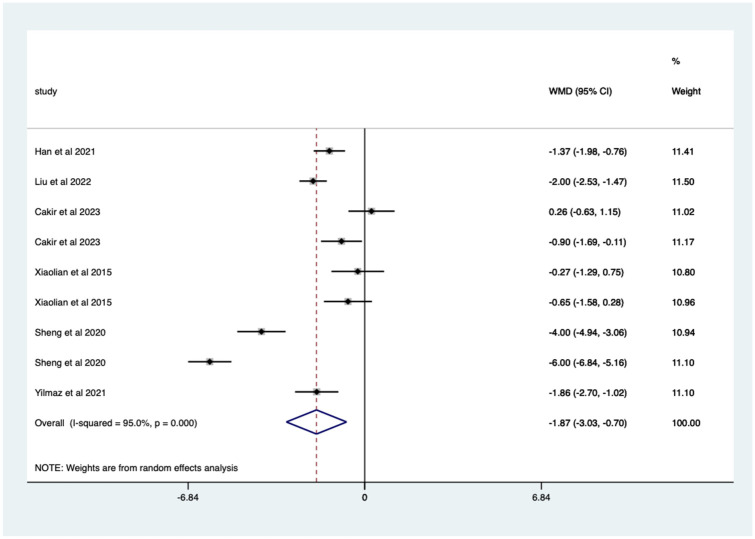
Studies with patients undergoing no sedation; pain score Forrest plot.

Studies with no sedation – significant mean 1.87 (CI 0.7–3.03) pain score reduction (Figure 14).

#### Anxiety

**Figure 15.**
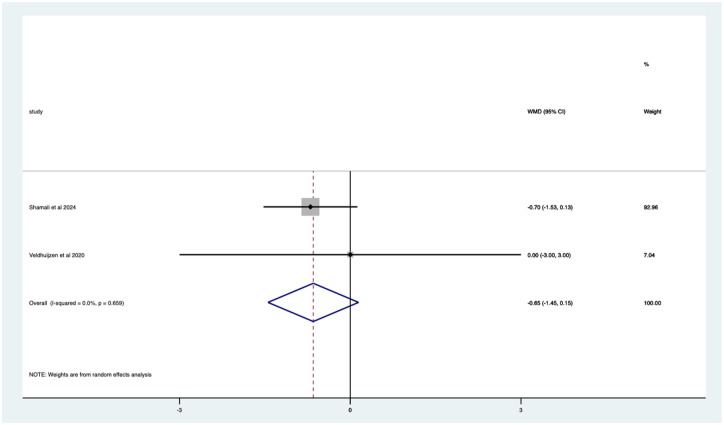
Studies with patients undergoing sedation; anxiety score Forrest plot.

**Figure 16.**
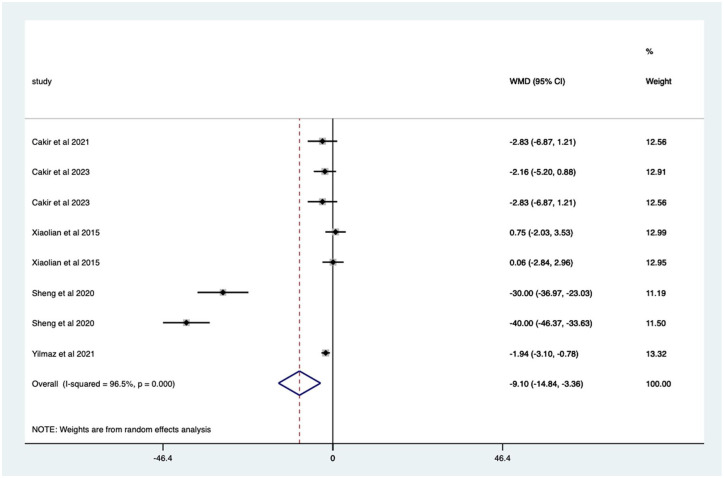
Studies with patients undergoing no sedation; anxiety score Forrest plot.

Studies with sedation – non-significant mean 0.65 (CI −0.15 to 1.45) anxiety score reduction (Figure 15). Studies with no sedation – significant mean 9.10 (CI 3.36–14.84) anxiety score reduction (Figure 16).

### Previous endotherapy

#### Pain

**Figure 17.**
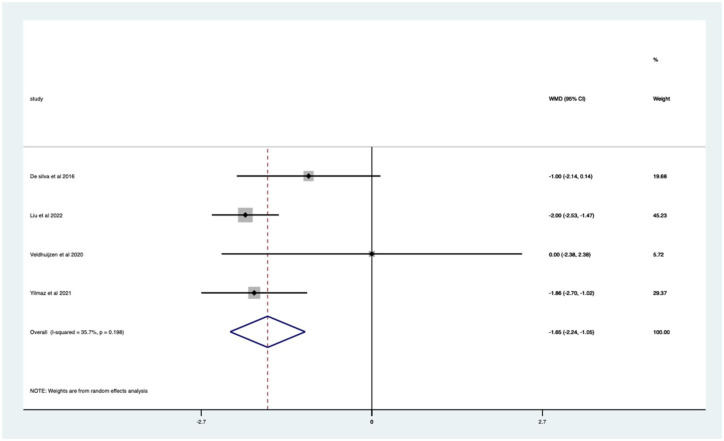
Studies with patients undergoing no endotherapy; pain score Forrest plot.

Studies with no endotherapy – significant mean 1.65 (CI 1.05–2.24) pain score reduction (Figure 17).

**Figure 18.**
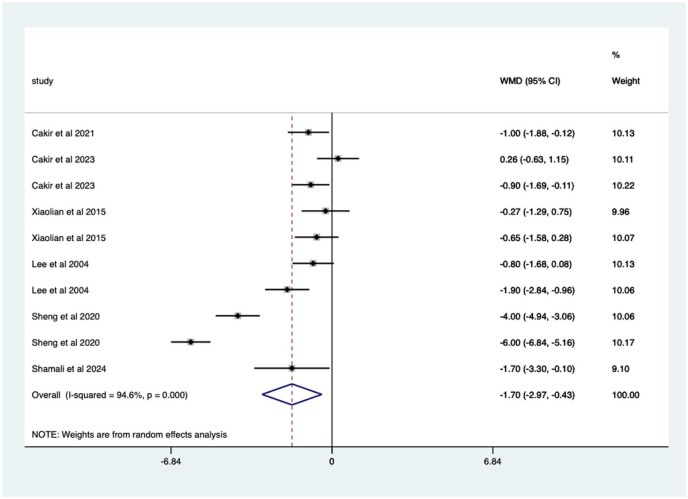
Studies with patients undergoing endotherapy; pain score Forrest plot.

Studies with endotherapy – significant mean 1.70 (CI 0.43–2.97) pain score reduction (Figure 18).

#### Anxiety

**Figure 19.**
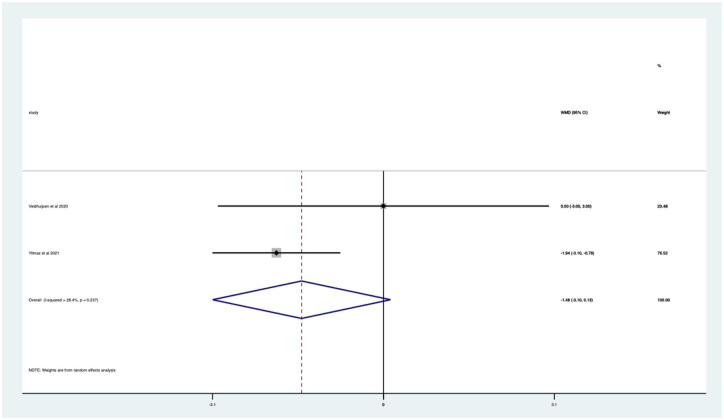
Studies with patients undergoing no endotherapy; anxiety score Forrest plot.

Studies with endotherapy – significant mean 10.54 (CI 2.36–18.71) anxiety score reduction. Studies with no endotherapy – non-significant mean 1.48 (CI −0.13 to 3.10) anxiety score reduction (Figure 19).

**Figure 20.**
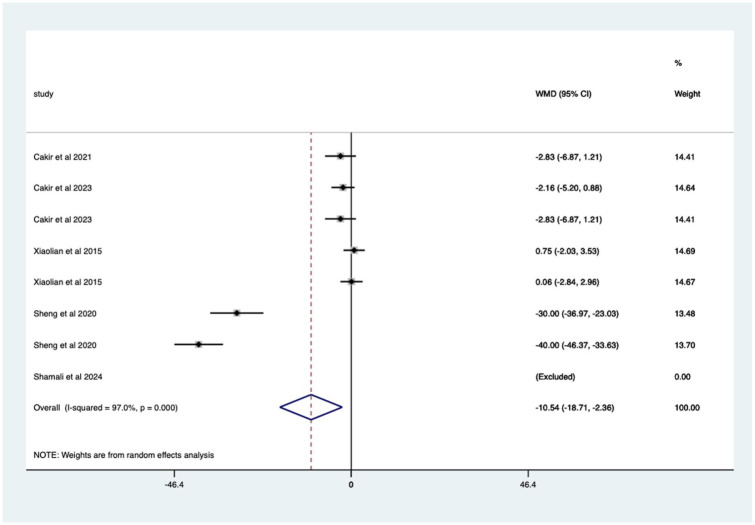
Studies with patients undergoing endotherapy; anxiety score Forrest plot.

Studies with endotherapy – significant mean 10.54 (CI 2.36–18.71) anxiety score reduction (Figure 20).

### Distraction type

#### Pain

**Figure 21.**
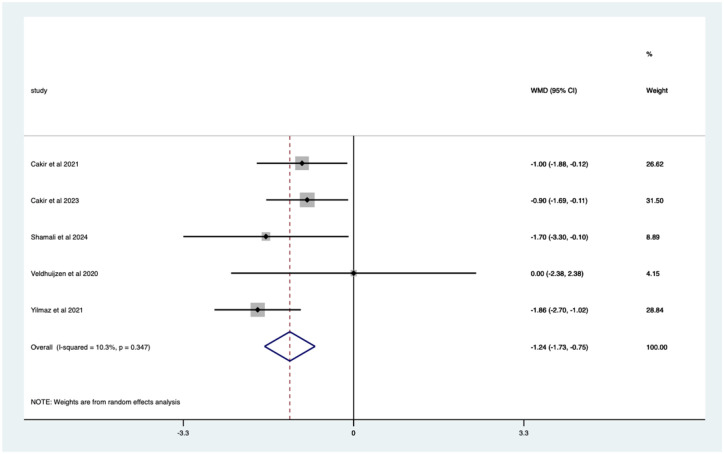
Studies with VR distraction; pain score Forrest plot. VR, virtual reality.

VR studies – significant mean 1.24 (CI 0.75–1.73) pain score reduction (Figure 21).

**Figure 22.**
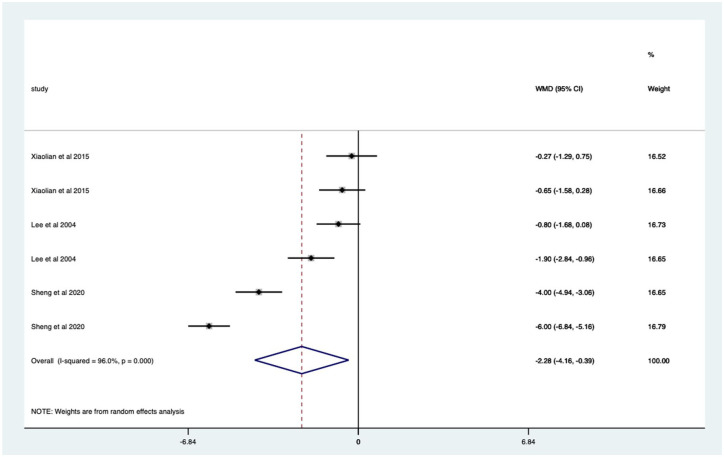
Studies with audio-visual distraction; pain score Forrest plot.

Audio-visual studies – significant mean 2.28 (CI 0.39–4.16) pain score reduction (Figure 22).

(Smartphone distraction studies did not have enough data to perform sub-analysis.)

#### Anxiety

**Figure 23.**
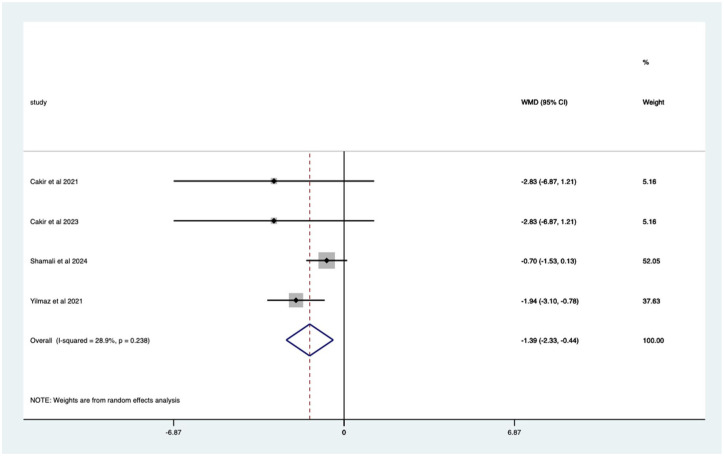
Studies with VR distraction; anxiety score Forrest plot. VR, virtual reality.

VR studies – significant mean 1.39 (CI 0.44–2.33) anxiety score reduction (Figure 23.

**Figure 24.**
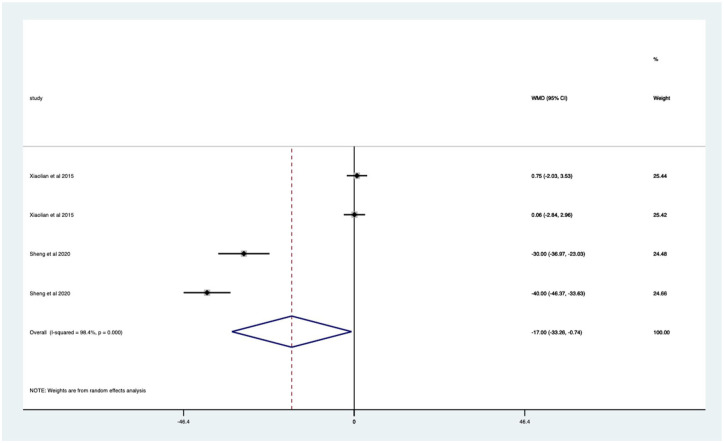
Studies with audio-visual distraction; anxiety score Forrest plot.

Audio-visual studies – significant mean 17 (CI 0.74–33.26) anxiety score reduction (Figure 24).

(Smartphone distraction studies did not have enough data to perform sub-analysis.)

### Publication bias assessment

**Figure 25.**
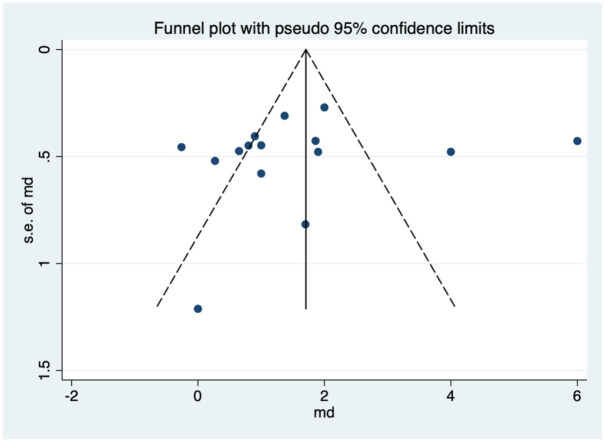
Publication bias funnel plot.

Egger’s regression test was performed on the study data with a non-significant result, suggesting no publication bias (*p* = 0.641). Figure 25 displays the funnel plot with full statistical analysis in Supplemental Appendix 5.

## Discussion

This systematic review and meta-analysis consisted of 27 RCT studies with 2753 patients. Overall outcomes for both pain and anxiety demonstrated a significant reduction in both scores using music and distraction techniques.

This paper has demonstrated the possible benefits of non-invasive, inexpensive interventions in the reduction of pain and anxiety scores. It has reinforced the evidence known on the more established technique of music and introduced task distraction as a comparable and similarly impactful means to improve pain and anxiety scores for patients.

The mean pain score reduction with music supports the findings of two previous meta-analyses.^4,23^ The mean anxiety score reduction with music also concurs with findings from Wang et al.^4^ The mean pain score reduction with distraction supports the findings of two recent meta-analyses.^25,26^ The mean anxiety score reduction with distraction is also in agreement with findings from Saab et al.^26^

A 1.5 and 1.59 pain score reduction represents a comparable 15%–16% decrease in pain reported by patients with the aid of intervention. This has the possibility of a significant clinical impact when studies have reported with sedation, a comparable decrease in pain of 10%.^49^ The clinical impact of the reduction in anxiety with these interventions has also been demonstrated, in particular with task distraction (7.49), where a score reduction between 5 and 10 is often considered meaningful.^50^

Alongside the two main primary outcomes analysed, other outcomes were identified to show the benefit from the intervention. Patients reported greater satisfaction, more willingness to undergo a repeat colonoscopy procedure, shorter duration of procedure, shorter caecal intubation time and lower average sedation use.^15–18,20,32–40,41–47^

Both interventions provide a low-cost and easy-to-implement option with potential scope to scale up with minimal or no adverse effects.^51^ Post-procedure care will also return at a lower cost compared to sedation.^52^ A strength of this meta-analysis is the wide array of studies included from western and eastern clinical settings ensuring wide representation of patient populations.

The sub-analysis of distraction techniques provided further supportive conclusions which can be inferred which may help improve pain and anxiety when implementing these interventions.

Blinding has been demonstrated to further strengthen the impact of distraction intervention for pain scores. This may be explained by the removal of any bias from both the endoscopist and patient, which may influence the impact of the intervention. Studies without blinding still reported a significant reduction in anxiety scores. A question left to answer is whether blinding will further improve the reduction of anxiety scores.

Patients who have undertaken a previous colonoscopy also demonstrated a greater impact from distraction. This may be as a result of previous experience with the procedure and the familiarity on the day, allowing the unknown aspects of an experience not to apply which will impact pain and anxiety.

Patients who did not undertake sedation compared to those who did reported a superior improvement in pain and anxiety with distraction techniques. This may be due to the patient being conscious and so with their cognition not being impaired, the distraction interventions were able to be more effective. Sedation is a confounding factor that should be highlighted, as this will have a knock-on effect on how music and distraction techniques are perceived by the patient, given the sedatory effect. It will also be a contributor to the reduction of pain and anxiety and therefore separating its effect from the interventions of music and distraction becomes more complex.

Patients who undertook endotherapy also reported a superior impact on pain and anxiety compared to those who did not. This may be due to a longer procedure, due to therapy being undertaken and therefore more time for the effect of the distraction technique to be applied. Audio-visual had a greater effect on pain and anxiety scores compared to visual alone and this is possibly due to the synergistic effect of both an audio and visual distraction modality being applied simultaneously.

There was a wide variability in study design for the music RCTs. The range of musical genres implemented was diverse and when patients were given a choice of music, this was demonstrated to be non-superior to pre-selected music by the study.^15,16,32–40^ The mode of music delivery varied between headphones and speaker output, with no clear method demonstrated to be significantly superior. The possible advantage of headphones is the ability to undertake blinding within the study. The disadvantage is reduced verbal communication with the patient, such as during position change requests. Speaker output for music could also prove distracting to the medical team. The duration of music or task distraction exposure was not explicitly outlined in all the studies, with the timing of music or distraction mostly commencing at the start of the procedure. Further analysis in this area may provide an optimum time to commence an intervention that has not been discovered. Two of the 11 music studies and 8 of the 12 distraction studies used no sedation and reported improvement in pain and anxiety, suggesting a positive impact that is not only reliant on sedative and analgesic medication.

### Sensitivity analysis

In this study, the subgroup analysis was used as the sensitivity analysis to ensure robust findings across the various patient cohorts. This was to address the *I*² values for all meta-analyses in this study, exceeding 80%, indicating substantial heterogeneity. Additionally, certain trials display exceptionally large effect sizes, and the sub-analysis has highlighted how this, however, does not significantly influence the overall results and therefore enhances the reliability of the study findings.

### Limitations

Some difficulties in the analysis included studies in which the STAI anxiety score scale varied in range and studies that did not explicitly provide scores and ranges of scores. Similar to previous meta-analyses undertaken with music, there was high heterogeneity present across the analysis which was also present for distraction. Possible explanations are regional variation, such as age and co-morbidities, methodology with music choice/distraction technique and lack of blinding in studies. The distribution from the funnel plot performed for publication bias (Figure 25) is reasonably symmetrical, with a few studies outside the funnel, which may suggest a minor publication bias or heterogeneity. Another limitation in the meta-analysis of distraction studies is the double use of controls to perform a pairwise comparison for RCTs with more than one intervention. This may affect the independent data and pooled estimates. However, the authors feel the conclusions drawn are still valid without this adding significantly to the heterogeneity and bias. A further limitation is where required missing data was imputed, such as the standard deviation during statistical analysis. The study quality was also impacted by the Jaded score for music, with few studies scoring 2 or less. The studies on distraction include a variety of distraction techniques and a question that is raised is the impact of distraction overall versus a specific type of distraction technique being utilised. Lastly, whilst an aim for low bias in the analysis was planned by using only RCT studies, the authors are aware of possible non-randomised studies that could have been utilised in addition to contribute to these findings.

Considerations for future studies include controlling for variables and taking the best performing information available from previous similar studies, such as music/distraction choice, duration, and delivery method. Potential areas of further research include exploring optimal duration and type of music or type of distraction technique. Future studies could also consider randomisation to be undertaken alongside blinding for the patient and endoscopist. The effect on supporting staff and the endoscopist themselves is an additional area to explore in future work, such as the effect of music on endoscopists and support staff in relation to concentration and relaxation. Finally, economic analysis of the scalability of these interventions will be crucial to add further evidence to the potential cost-saving benefits and implementation capability.

## Conclusion

This systematic review and meta-analysis have demonstrated a potential role for music and task distraction to improve pain and anxiety for patients undergoing a colonoscopy. The use of such interventions can be in addition to or exclusively utilised during a colonoscopy. It also provides an alternative safe option for patients who may not be eligible for sedation. Whilst evidence is still not directly comparable, it is plausible for the claim of establishing in daily clinical practice.

This meta-analysis utilising studies from both western and eastern cultures allows a better understanding to develop tools and methods to influence pain and anxiety for all patient groups, taking into consideration all cultural differences. In addition, the heterogeneity reflects daily practice with similarities in varying cultures and patient demographics that will be encountered in real-world applications.

As there are no comparative studies looking at task distraction versus music intervention, in the absence of a trial which formally randomises the three arms of task distraction, music and control, this meta-analysis has reported uniquely the nearest results from the data that reflect real-world practice.

Future recommendations to strengthen future studies include increasing patient recruitment to ensure adequate power and decreasing the risk of bias, controlling for variables that may have a confounding factor on results, such as music choice, sedation choice and dosage, and finally conducting studies in a randomised manner with blinding.

## Supplemental Material

sj-docx-1-tag-10.1177_17562848251378236 – Supplemental material for The effect of music and distraction on pain and anxiety during colonoscopy: a systematic review and meta-analysis Supplemental material, sj-docx-1-tag-10.1177_17562848251378236 for The effect of music and distraction on pain and anxiety during colonoscopy: a systematic review and meta-analysis by Jabed F. Ahmed, Hutan Ashrafian, Ara Darzi, Ferdinando R. Baena and Nisha Patel in Therapeutic Advances in Gastroenterology

sj-docx-2-tag-10.1177_17562848251378236 – Supplemental material for The effect of music and distraction on pain and anxiety during colonoscopy: a systematic review and meta-analysis Supplemental material, sj-docx-2-tag-10.1177_17562848251378236 for The effect of music and distraction on pain and anxiety during colonoscopy: a systematic review and meta-analysis by Jabed F. Ahmed, Hutan Ashrafian, Ara Darzi, Ferdinando R. Baena and Nisha Patel in Therapeutic Advances in Gastroenterology

sj-docx-3-tag-10.1177_17562848251378236 – Supplemental material for The effect of music and distraction on pain and anxiety during colonoscopy: a systematic review and meta-analysis Supplemental material, sj-docx-3-tag-10.1177_17562848251378236 for The effect of music and distraction on pain and anxiety during colonoscopy: a systematic review and meta-analysis by Jabed F. Ahmed, Hutan Ashrafian, Ara Darzi, Ferdinando R. Baena and Nisha Patel in Therapeutic Advances in Gastroenterology

sj-docx-4-tag-10.1177_17562848251378236 – Supplemental material for The effect of music and distraction on pain and anxiety during colonoscopy: a systematic review and meta-analysis Supplemental material, sj-docx-4-tag-10.1177_17562848251378236 for The effect of music and distraction on pain and anxiety during colonoscopy: a systematic review and meta-analysis by Jabed F. Ahmed, Hutan Ashrafian, Ara Darzi, Ferdinando R. Baena and Nisha Patel in Therapeutic Advances in Gastroenterology

## Declarations

**Ethical approval and consent to participate:** Not applicable.

**Consent for publication:** All authors give consent for publication.

**Availability of data and materials:** All information available on request.

## References

[1] Triantafillidis JK Vagianos C Malgarinos G. Colonoscopy in colorectal cancer screening: current aspects. Indian J Surg Oncol 2015; 6: 237–250. 27217671 10.1007/s13193-015-0410-3 PMC4856683

[2] Nierengarten MB. Colonoscopy remains the gold standard for screening despite recent tarnish. Cancer 2023; 129: 330–331. 36602936 10.1002/cncr.34622

[3] Ryhlander J Ringström G Lindkvist B , et al. Risk factors for underestimation of patient pain in outpatient colonoscopy. Scand J Gastroenterol 2022; 57: 1120–1130. 35486038 10.1080/00365521.2022.2063034

[4] Wang MC Zhang LY Zhang YL , et al. Effect of music in endoscopy procedures: systematic review and meta-analysis of randomized controlled trials. Pain Med 2014; 15: 1786–1794. 25139786 10.1111/pme.12514

[5] Bechtold ML Puli SR Othman MO , et al. Effect of music on patients undergoing colonoscopy: a meta-analysis of randomized controlled trials. Dig Dis Sci 2009; 54: 19–24. 18483858 10.1007/s10620-008-0312-0

[6] Ghevariya V Duddempudi S Ghevariya N , et al. Barriers to screening colonoscopy in an urban population: a study to help focus further efforts to attain full compliance. Int J Colorectal Dis 2013; 28: 1497–1503. 23666513 10.1007/s00384-013-1708-7

[7] Sidhu R Turnbull D Haboubi H , et al. British Society of Gastroenterology guidelines on sedation in gastrointestinal endoscopy. Gut 2024; 73: 219–245. 37816587 10.1136/gutjnl-2023-330396 PMC10850688

[8] Ball AJ Campbell JA Riley SA. Nitrous oxide use during colonoscopy: a national survey of English screening colonoscopists. Frontline Gastroenterol 2014; 5: 254–259. 28839782 10.1136/flgastro-2014-100446 PMC5369751

[9] Patiyal N Kalyani V Mishra R , et al. Effect of music therapy on pain, anxiety, and use of opioids among patients underwent orthopedic surgery: a systematic review and meta-analysis. Cureus 2021; 13: e18377. 10.7759/cureus.18377 PMC8555445 34725621

[10] Yinger OS Gooding LF. A systematic review of music-based interventions for procedural support. J Music Ther 2015; 52: 1–77. 25878063 10.1093/jmt/thv004

[11] Klassen JA Liang Y Tjosvold L , et al. Music for pain and anxiety in children undergoing medical procedures: a systematic review of randomized controlled trials. Ambul Pediatr 2008; 8: 117–128. 18355741 10.1016/j.ambp.2007.12.005

[12] Trappe HJ. Music and health – what kind of music is helpful for whom? What music not? Dtsch Med Wochenschr 2009; 134: 2601–2606. 20013543 10.1055/s-0029-1243066

[13] Miluk-Kolasa B Obminski Z Stupnicki R , et al. Effects of music treatment on salivary cortisol in patients exposed to pre-surgical stress. Exp Clin Endocrinol 1994; 102: 118–120. 8056055 10.1055/s-0029-1211273

[14] Mojtabavi H Saghazadeh A Valenti VE , et al. Can music influence cardiac autonomic system? A systematic review and narrative synthesis to evaluate its impact on heart rate variability. Complement Ther Clin Pract 2020; 39: 101–162. 10.1016/j.ctcp.2020.101162 32379689

[15] Cakir SK Evirgen S. Three distraction methods for pain reduction during colonoscopy: a randomized controlled trial evaluating the effects on pain and anxiety. J Perianesth Nurs 2023; 38: 1–7. 37565937 10.1016/j.jopan.2023.02.007

[16] De Silva AP Niriella MA Nandamuni Y , et al. Effect of audio and visual distraction on patients undergoing colonoscopy: a randomized controlled study. Endosc Int Open 2016; 4: 1211–1214. 10.1055/s-0042-117630 PMC5110335 27853748

[17] Xiaolian J Xiaolin L Lan ZH. Effects of visual and audiovisual distraction on pain and anxiety among patients undergoing colonoscopy. Gastroenterol Nurs 2015; 38: 55–61. 25636013 10.1097/SGA.0000000000000089

[18] Lee DW Chan AC Wong SK , et al. Can visual distraction decrease the dose of patient-controlled sedation required during colonoscopy? A prospective randomized controlled trial. Endoscopy 2004; 36: 197–201. 14986215 10.1055/s-2004-814247

[19] Sheng LP Han CQ Nie C , et al. Watching videos of colonoscopies and receiving interpretations reduce pain and anxiety while increasing the satisfaction of patients. Dig Dis Sci 2021; 66: 541–546. 32193861 10.1007/s10620-020-06186-6

[20] Garrett B Taverner T McDade P. Virtual reality as an adjunct home therapy in chronic pain management: an exploratory study. JMIR Med Inform 2017; 5(2): e11. 10.2196/medinform.7271 PMC5445235 28495661

[21] Rudin D Kiss A Wetz RV , et al. Music in the endoscopy suite: a meta-analysis of randomized controlled studies. Endoscopy 2007; 39: 507–510. 17554644 10.1055/s-2007-966362

[22] Tam WW Wong EL Twinn SF. Effect of music on procedure time and sedation during colonoscopy: a meta-analysis. World J Gastroenterol 2008; 14: 5336–5343. 18785289 10.3748/wjg.14.5336 PMC2744067

[23] Heath RD Parsa N Matteson-Kome ML , et al. Use of music during colonoscopy: an updated meta-analysis of randomized controlled trials. World J Meta-Anal 2019; 7: 428–435.

[24] Sorkpor SK Johnson CM Santa Maria DM , et al. The effect of music listening on pain in adults undergoing colonoscopy: a systematic review and meta-analysis. J Perianesth Nurs 2021; 36: 573–580. 33994100 10.1016/j.jopan.2020.12.012

[25] Zhang YY Vimala R Chui PL , et al. Effect of visual distraction on pain in adults undergoing colonoscopy: a meta-analysis. Surg Endosc 2023; 37(4): 2633–2643. 36369410 10.1007/s00464-022-09724-7

[26] Saab O Al-Obaidi H Merza N , et al. The impact of visual distraction interventions on patients’ pain and anxiety during colonoscopy: a systematic review and meta-analysis of randomized controlled trials. J Clin Gastroenterol 2025; 59: 849–862. 39495815 10.1097/MCG.0000000000002086

[27] PRISMA Statement [Internet]. [cited 2020], http://prisma-statement.org/PRISMAStatement/PRISMAStatement (2009, accessed 12 August 2025).

[28] Harikumar R Raj M Paul A , et al. Listening to music decreases need for sedative medication during colonoscopy: a randomized, controlled trial. Indian J Gastroenterol 2006; 25: 3–5. 16567885

[29] Schiemann U Gross M Reuter R , et al. Improved procedure of colonoscopy under accompanying music therapy. Eur J Med Res 2002; 7: 131–134. 11953285

[30] Price DD McGrath PA Rafii A , et al. The validation of visual analogue scales as ratio scale measures for chronic and experimental pain. Pain 1983; 17: 45–56. 6226917 10.1016/0304-3959(83)90126-4

[31] Spielberger CD Gorsuch RL Lushene R , et al. Manual for the State-Trait Anxiety Inventory. Palo Alto, CA: Consulting Psychologists Press.

[32] Çelebi D Yılmaz E Şahin ST , et al. The effect of music therapy during colonoscopy on pain, anxiety and patient comfort: a randomized controlled trial. Complement Ther Clin Pract 2020; 38: 101084. 32056820 10.1016/j.ctcp.2019.101084

[33] Ovayolu N Ucan O Pehlivan S , et al. Listening to Turkish classical music decreases patients’ anxiety, pain, dissatisfaction and the dose of sedative and analgesic drugs during colonoscopy: a prospective randomized controlled trial. World J Gastroenterol 2006; 12: 7532–7536. 17167846 10.3748/wjg.v12.i46.7532 PMC4087603

[34] Binek J Sagmeister M Borovicka J , et al. Perception of gastrointestinal endoscopy by patients and examiners with and without background music. Digestion 2003; 68: 5–8. 12949433 10.1159/000073219

[35] Ko CH Chen YY Wu KT , et al. Effect of music on level of anxiety in patients undergoing colonoscopy without sedation. J Chin Med Assoc 2017; 80: 154–160. 27889459 10.1016/j.jcma.2016.08.010

[36] Lee DW Chan KW Poon CM , et al. Relaxation music decreases the dose of patient-controlled sedation during colonoscopy: a prospective randomized controlled trial. Gastrointest Endosc 2002; 55: 33–36. 11756911 10.1067/mge.2002.120387

[37] Bechtold ML Perez RA Puli SR , et al. Effect of music on patients undergoing outpatient colonoscopy. World J Gastroenterol 2006; 12: 7309–7312. 17143946 10.3748/wjg.v12.i45.7309 PMC4087488

[38] Brix LD Pedersen ASB . Effect of music intervention in colonoscopy-naïve adults: a randomised controlled trial. Br J Nurs 2022; 31: 526–532. 35648674 10.12968/bjon.2022.31.10.526

[39] Costa A Montalbano LM Orlando A , et al. Music for colonoscopy: a single-blind randomized controlled trial. Dig Liver Dis 2010; 42: 871–876. 20452299 10.1016/j.dld.2010.03.016

[40] Martindale F Mikocka-Walus AA Walus BP , et al. The effects of a designer music intervention on patients’ anxiety, pain, and experience of colonoscopy: a short report on a pilot study. Gastroenterol Nurs 2014; 37: 338–342. 25271826 10.1097/SGA.0000000000000066

[41] Han C Xu T Sheng L , et al. Improving the discomfort and satisfaction of colonoscopy by distraction with smartphones: a prospective randomized controlled study. Medicine (Baltimore) 2021; 100: e23799. 10.1097/MD.0000000000023799 PMC7939225 33655906

[42] Karaveli Çakır S Evirgen S . The effect of virtual reality on pain and anxiety during colonoscopy: a randomized controlled trial. Turk J Gastroenterol 2021; 32: 451–457. 34231475 10.5152/tjg.2021.191081 PMC8975482

[43] Liu Q Zang Y Zang W , et al. Implementation of virtual reality technology to decrease patients’ pain and nervousness during colonoscopies: a prospective randomised controlled single-blinded trial. Clin Med (Lond) 2022; 22: 237–240. 35443967 10.7861/clinmed.2022-0001 PMC9135071

[44] Umezawa S Higurashi T Uchiyama S , et al. Visual distraction alone for the improvement of colonoscopy-related pain and satisfaction. World J Gastroenterol 2015; 21: 4707–4714. 25914482 10.3748/wjg.v21.i15.4707 PMC4402320

[45] Shamali M Vilmann P Johansen NR , et al. Virtual reality intervention to improve quality of care during colonoscopy: a hybrid type 1 randomized controlled trial. Gastrointest Endosc 2024; 100(5): 914–922.e2. 10.1016/j.gie.2024.05.023 38851457

[46] Veldhuijzen G Klaassen NJM Van Wezel RJA , et al. Virtual reality distraction for patients to relieve pain and discomfort during colonoscopy. Endosc Int Open 2020; 8(7): E959–E966. 10.1055/a-1178-9289 PMC7326580 32626819

[47] Doğan Yılmaz E Ünlüsoy Dinçer N . The effects of virtual reality glasses on vital signs and anxiety in patients undergoing colonoscopy: a randomized controlled trial. Gastroenterol Nurs 2023; 46(4): 318–328. 37278621 10.1097/SGA.0000000000000733

[48] Jadad A . Jadad scale for reporting randomised control trials [Internet]. [Cited 2005], https://onlinelibrary.wiley.com/doi/pdf/10.1002/9780470988343.app1 (1996, accessed 12 August 2025).

[49] Aljebreen AM Almadi MA Leung FW. Sedated vs unsedated colonoscopy: a prospective study. World J Gastroenterol 2014; 20(17): 5113–5118. 24803827 10.3748/wjg.v20.i17.5113 PMC4009549

[50] Karpuzcu HC Yarbaş G Çatalbaş R , et al. The effect of pre-procedural anxiety level on the quality of upper GI endoscopy in non-sedated patients: “can the need for sedation be predicted?.” Therap Adv Gastroenterol 2025; 18: 17562848251333025. 10.1177/17562848251333025 PMC12034957 40297209

[51] Spagnuolo R Corea A Blumetti M , et al. Effects of listening to music in digestive endoscopy: a prospective intervention study led by nursing. J Adv Nurs 2020; 76(11): 2993–3002. 32901972 10.1111/jan.14516

[52] Lin OS. Sedation for routine gastrointestinal endoscopic procedures: a review on efficacy, safety, efficiency, cost and satisfaction. Intest Res 2017; 15(4): 456–466. 29142513 10.5217/ir.2017.15.4.456 PMC5683976

