# Supplementary material for: The effect of music and distraction on pain and anxiety during colonoscopy: a systematic review and meta-analysis
Source: Ther Adv Gastroenterol. 2025 Oct 2;18:17562848251378236. doi: 10.1177/17562848251378236 (PMC12491824; doi:10.1177/17562848251378236)
Supplement: sj-docx-3-tag-10.1177_17562848251378236 – Supplemental material for The effect of music and distraction on pain and anxiety during colonoscopy: a systematic review and meta-analysis [file sj-docx-3-tag-10.1177_17562848251378236.docx]

**Appendices 1**

**Meta-Analysis and Systematic Review - Literature Review protocol**

1. **Aims:**

The literature search aims to evaluate the current evidence available in the use of music and task distraction and its potential to improve pain and discomfort in colonoscopy.

1. **Search questions:**

| Question 1 | | MeSH terms |
| --- | --- | --- |
| **Population** | GI endoscopy (simulated or human) | Endoscopy, Esophagogastroduodenoscopy, Oesophagogastroduodenoscopy, Colonoscopy, Sigmoidoscopy, Capsule Endoscopy. |
| **Intervention** | Music, Task distraction | music, task, smartphone, audio, video, distraction, virtual reality |
| **Comparator group** | standard colonoscopy |  |
| **Outcome** | Pain and discomfort/ systematic review of all available evidence |  |

### **Information sources of literature:**

The following clinical databases will be searched:

1. **Medline:** https://www.ncbi.nlm.nih.gov/pubmed/
2. **Embase:** https://www.elsevier.com/en-gb/products/embase/content
3. **Cochrane Reviews:** https://www.cochranelibrary.com
4. **Google Scholar:** https://scholar.google.com/

### **Inclusion / Exclusion criteria**

Retrieved literature will be selected according to the inclusion / exclusion criteria given below:

| **Code** | **Inclusion criteria** |
| --- | --- |
| I1 | Adult patients (Age 18 and above) |
| I2 | Peer reviewed publications (Randomised Control Trial) |
| I3 | Literature published in the English language |
| I4 | Relevant to question |
| I5 | Colonoscopy procedure |

| **Code** | **Exclusion criteria** |
| --- | --- |
| E1 | Duplicate publications or no original data present |
| E2 | No full text available |
| E2 | Literature published in a language other than English |
| E3 | Not relevant to question |
| E4 | Gastroscopy or Sigmoidoscopy procedure |

1. **Selection process:**

Two independent reviewers each separately screened each paper.

RISK OF BIAS ASSESSMENT – The two reviewers assessed the title and abstract separately with the decision to include a paper for the next stage only being possible if both reviewers chose to include on their individual assessment. If only one reviewer deemed the paper acceptable this would not qualify for the next stage of the literature review.

Data collection process:

No automated tools were used for the literature collection. Papers were manually listed and checked against inclusion and exclusion criteria with results input into a table.

Synthesis methods:

Spreadsheet software was used to collect data from each study. For each study the following was extracted: (1) study details of authors, year of publication and journal; (2) study design and if RCT; (3) mean pain score or mean anxiety score (scale used, range, standard deviation or interquartile range provided)

Data was input in tabulated form of individual results that were needed such as visual analogue score, STAI anxiety score

1. **Search results review:**

Databases will be searched using Medical Search Headings (MeSH) where possible:

**MEDLINE:**

**Music**

- - - 1. **Music – 16922**
      2. **Colonoscopy - 35182**
      3. **Music AND Colonoscopy – 10**
      4. **Music AND colonoscopy AND pain – 2**

**Smartphone**

**Smartphone – 9309**

**Colonoscopy – 35182**

**Smartphone AND colonoscopy – 14**

**Smartphone AND distraction AND colonoscopy – 1**

**Virtual reality**

1. **Virtual reality – 5893**
2. **Colonoscopy – 35182**
3. **VR AND colonoscopy – 8**

**Task Distraction**

**nothing on task distraction that’s not smartphone**

**Distraction**

1. **Distraction – not a MeSH term**
2. **Distraction AND colonoscopy – 32**

**EMBASE:**

**Music**

- - - 1. **Music AND Colonoscopy – 9**
      2. **Music AND colonoscopy AND pain – 2**

**Smartphone**

**Smartphone AND colonoscopy – 14**

**Smartphone AND distraction AND colonoscopy – 2**

**Virtual reality**

**VR AND colonoscopy – 8**

**Task Distraction**

**nothing on task distraction that’s not smartphone**

**Distraction**

**Distraction AND colonoscopy – 12**

**COCHRANE REVIEW:**

**Music**

1. **Music – 1130**
2. **Colonoscopy - 3115**
3. **Music AND Colonoscopy – 10 (all trials, no reviews)**

**Smartphone**

**Smartphone – 1040**

**Colonoscopy – 3115**

**Smartphone AND colonoscopy – 14 (all trials, no reviews)**

**Virtual reality**

1. **Virtual reality – 1012**
2. **Colonoscopy – 3115**
3. **VR AND colonoscopy – 8 (all trials, no reviews)**

**Task Distraction**

**nothing on task distraction that’s not smartphone**

**Distraction**

1. **Distraction – 16**
2. **Colonoscopy - 3115**
3. **Distraction AND colonoscopy – 0**

**GOOGLE SCHOLAR**

**Music**

**Music AND Colonoscopy – 9**

**Smartphone**

**Smartphone AND colonoscopy – 0**

**Smartphone AND distraction AND colonoscopy – 0**

**Virtual reality**

**VR AND colonoscopy – 5**

**Task Distraction**

*nothing on task distraction that’s not smartphone*

**Distraction**

**Distraction – not a MeSH term**

**Distraction AND colonoscopy – 32**

**Search Summary:**

**
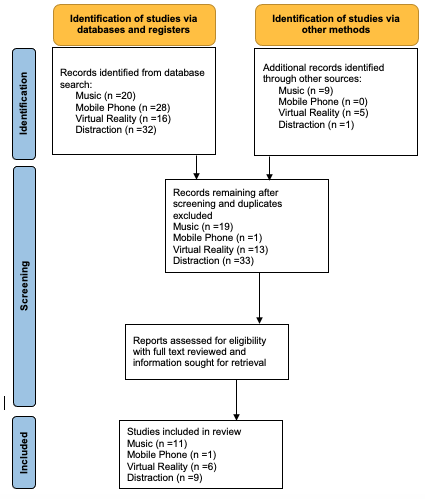
**

**Studies to assess further:**

- Music – 11 studies
- Smartphone – 1 study
- Virtual reality – 6 studies
- Distraction – 9 studies

1. De Silva AP, Niriella MA, Nandamuni Y, et al. Effect of audio and visual distraction on patients undergoing colonoscopy: a randomized controlled study. *Endosc Int Open* 2016; 4: 1211-1214.
2. Cakir SK, Evirgen S. Three Distraction Methods for Pain Reduction During Colonoscopy: A Randomized Controlled Trial Evaluating the Effects on Pain and Anxiety*. J Perianesth Nurs* 2023; 38: 1-7.
3. Çelebi D, Yılmaz E, Şahin ST, Baydur H. The effect of music therapy during colonoscopy on pain, anxiety and patient comfort: A randomized controlled trial. *Complement Ther Clin Pract* 2020; 38: 101084.
4. Costa A, Montalbano LM, Orlando A, et al. Music for colonoscopy: A single-blind randomized controlled trial. *Dig Liver Dis* 2010; 42: 871-876.
5. Martindale F, Mikocka-Walus AA, Walus BP, Keage H, Andrews JM. The effects of a designer music intervention on patients' anxiety, pain, and experience of colonoscopy: a short report on a pilot study. *Gastroenterol Nurs* 2014; 37: 338-342.
6. Ovayolu N, Ucan O, Pehlivan S, et al. Listening to Turkish classical music decreases patients' anxiety, pain, dissatisfaction and the dose of sedative and analgesic drugs during colonoscopy: a prospective randomized controlled trial. *World J Gastroenterol* 2006; 12: 7532-7536.
7. Bechtold ML, Perez RA, Puli SR, Marshall JB. Effect of music on patients undergoing outpatient colonoscopy. *World J Gastroenterol* 2006; 12: 7309-7312.
8. Lee DW, Chan KW, Poon CM, et al. Relaxation music decreases the dose of patient-controlled sedation during colonoscopy: a prospective randomized controlled trial. *Gastrointest Endosc* 2002; 55: 33-36.
9. Brix LD, Pedersen ASB. Effect of music intervention in colonoscopy-naïve adults: a randomised controlled trial. *Br J Nurs* 2022; 31: 526-532.
10. Binek J, Sagmeister M, Borovicka J, Knierim M, Magdeburg B, Meyenberger C. Perception of gastrointestinal endoscopy by patients and examiners with and without background music. *Digestion* 2003; 68: 5-8.
11. Ko CH, Chen YY, Wu KT, et al. Effect of music on level of anxiety in patients undergoing colonoscopy without sedation. *J Chin Med Assoc* 2017; 80: 154-160.
12. Xiaolian J, Xiaolin L, Lan ZH. Effects of visual and audiovisual distraction on pain and anxiety among patients undergoing colonoscopy. *Gastroenterol Nurs* 2015; 38: 55-61.
13. Lee DW, Chan AC, Wong SK, et al. Can visual distraction decrease the dose of patient-controlled sedation required during colonoscopy? A prospective randomized controlled trial. *Endoscopy* 2004; 36: 197-201.
14. Sheng LP, Han CQ, Nie C, et al. Watching Videos of Colonoscopies and Receiving Interpretations Reduce Pain and Anxiety While Increasing the Satisfaction of Patients. *Dig Dis Sci* 2021; 66: 541-546.
15. Karaveli Çakır S, Evirgen S. The Effect of Virtual Reality on Pain and Anxiety During Colonoscopy: A Randomized Controlled Trial. *Turk J Gastroenterol* 2021; 32: 451-457.
16. Liu Q, Zang Y, Zang W, et al. Implementation of virtual reality technology to decrease patients' pain and nervousness during colonoscopies: a prospective randomised controlled single-blinded trial. *Clin Med (Lond)* 2022; 22: 237-240.
17. Umezawa S, Higurashi T, Uchiyama S, et al. Visual distraction alone for the improvement of colonoscopy-related pain and satisfaction. *World J Gastroenterol* 2015; 21: 4707-4714.
18. Shamali M, Vilmann P, Johansen NR et al. Virtual reality intervention to improve quality of care during colonoscopy: a hybrid type 1 randomized controlled trial. *Gastrointest Endosc*. 2024;100(5):914-922.e2.
19. Veldhuijzen G, Klaassen NJM, Van Wezel RJA et al. Virtual reality distraction for patients to relieve pain and discomfort during colonoscopy. *Endosc Int Open*. 2020;8(7):E959-E966.
20. Doğan Yılmaz E, Ünlüsoy Dinçer N. The Effects of Virtual Reality Glasses on Vital Signs and Anxiety in Patients Undergoing Colonoscopy: A Randomized Controlled Trial. *Gastroenterol Nurs*. 2023;46(4):318-328.
21. **Data extraction from full review:**

Study characteristics from music RCTs

| **Study** | **Type of Music** | **How music played** | **Blinding** | **Duration** | **Use of sedation** | **No of patients (control/intervention)** | **Findings (intervention vs control)** |
| --- | --- | --- | --- | --- | --- | --- | --- |
| **Lee et al 2002^32^** | Classical, jazz, popular (Chinese or English language), and Chinese opera | Headphones | Yes | Not specified | Group 2 – PCA only  Group 3 - Music only group could request sedation | 110 (55/55) | Pain - nonsignificant reduction |
| **Binek et al 2003^29^** | Multiple styles of music (non-classical/ light or classical). | Speaker | No | Not specified | Used as per patient request | 301 (150/151) | Pain - significant reduction |
| **Bechtold et al 2006^33^** | Album titled “Watermark” by Enya | Speaker | Yes | Not specified | All sedated | 166 (81/85) | Pain and Anxiety -nonsignificant reduction |
| **Ovayolu et al 2006^28^** | Turkish | Speaker | No | Before and during procedure | Sedation used. Number of patients not specified | 60 (30/30) | Pain and Anxiety -significant reduction |
| **Costa et al 2010^35^** | Blues, swing, classic, country, jazz, glam-rock, 50–60–70s rock, instrumental, new age, Celtic, reggae, relaxing, Spanish music, classic Italian pop, modern Italian pop, classic American pop, motion picture sound, native American. | Headphones | Yes | Not specified | Used as per patient request. Midazolam and Pethidine. | 109 (53/56) | Pain - significant reduction |
| **Martindale et al 2014^36^** | Johann Sebastian Bach (Classical) | Headphones | No | During procedure | Sedation used. Number of patients not specified | 34 (17/17) | Pain and Anxiety -nonsignificant reduction |
| **De Silva et al 2016^31^** | Sinhala, Hindi, Classic, and Hip-hop genres | Head mounted set | Yes | During procedure | All sedated. Midazolam and Pethidine | 133 (67/66) | Pain - significant reduction |
| **Ko et al 2017^30^** | Classical music or light music | Speaker (from Ipad) | No | During procedure | No sedation | 138 (57/81) | Anxiety - significant reduction |
| **Celebi et al 2020^27^** | Turkish classical | Headphones | No | During procedure | All sedated. Midazolam 2mg | 112 (56/56) | Pain and Anxiety -significant reduction |
| **Brix et al 2022^34^** | instrumental acoustic music with integrated sounds of nature, intended to form a connection with everyone no matter their taste in music, listening habits and preferences. | Headphones 30 minutes before and then music pillow during procedure | No | Before and during procedure | Used as per patient request. | 337 (168/169) | Pain and Anxiety -nonsignificant reduction |
| **Cakir et al 2023^17^** | Acemasiran-type classical Turkish music | Headphones | No | Before and during procedure | No sedation | 60 (30/30) | Pain - significant reduction  Anxiety - nonsignificant reduction |

Study characteristics from distraction RCTs

| **Study** | **Type of distraction** | **Method of delivery** | **blinding** | **No of patients (control/intervention)** | **Use of sedation** | **Findings (intervention vs control)** |
| --- | --- | --- | --- | --- | --- | --- |
| **De Silva et al 2016^16^** | Visual | Movie viewed on screen | Yes | 134 (67/67) | All sedated. Midazolam and Pethidine | Pain - significant reduction  Anxiety - nonsignificant reduction |
| **Han et al 2021^37^** | Smartphone | Not specified | No | 360 (180/180) | none | Pain - significant reduction |
| **Cakir et al 2021^38^** | Virtual Reality | Headset | No | 60 (30/30) | None | Pain - significant reduction  Anxiety - nonsignificant reduction |
| **Liu et al 2022^39^** | Virtual Reality | Headset | Yes | 120 (60/60) | None | Pain - significant reduction |
| **Cakir et al 2023^17^** | Stress Ball | - | No | 60 (30/30) | None | Pain - significant reduction  Anxiety - nonsignificant reduction |
| **Cakir et al 2023^17^** | Virtual Reality | Headset | No | 60 (30/30) | None | Pain - significant reduction  Anxiety - nonsignificant reduction |
| **Umezawa et al 2015^40^** | Video | Head mounted display | Yes | 60 (30/30) | None | Pain and anxiety- nonsignificant reduction |
| **Xiaolian et al 2015^18^** | Visual | Soundless DVD with earphones | No | 120 (60/60) | None | Pain - significant reduction  Anxiety - nonsignificant reduction |
| **Xiaolian et al 2015^18^** | Audiovisual | DVD with earphones | No | 120 (60/60) | None | Pain - significant reduction  Anxiety - nonsignificant reduction |
| **Lee et al 2004^41^** | Visual | Headset | No | 105 (53/52) | All sedated. Propofol and Alfentanil | Pain - significant reduction |
| **Lee et al 2004^41^** | Audiovisual | Headset | No | 105 (53/52) | All sedated. Propofol and Alfentanil | Pain - significant reduction |
| **Sheng et al 2020^20^** | Video | Headset | No | 120 (60/60) | None | Pain and Anxiety -significant reduction |
| **Sheng et al 2020^20^** | Audiovisual | Headset | No | 120 (60/60) | None | Pain and Anxiety -significant reduction |
| **Shamali et al 2024^42^** | Virtual Reality | Headset | No | 47 (24/23) | Midazolam and Fentanyl 30 patients from both groups. | Pain – significant reduction  Anxiety - nonsignificant reduction |
| **Veldhuijzen et al 2020^43^** | Virtual Reality | Headset | No | 19 (9/10) | Midazolam and alfentanyl. | Pain and anxiety – non significant reduction |
| **Yilmaz et al 2021^44^** | Virtual Reality | Headset | No | 44 (22/22) | None | Pain and Anxiety -significant reduction |
