# Supplementary material for: The effect of music and distraction on pain and anxiety during colonoscopy: a systematic review and meta-analysis
Source: Ther Adv Gastroenterol. 2025 Oct 2;18:17562848251378236. doi: 10.1177/17562848251378236 (PMC12491824; doi:10.1177/17562848251378236)
Supplement: sj-docx-4-tag-10.1177_17562848251378236 – Supplemental material for The effect of music and distraction on pain and anxiety during colonoscopy: a systematic review and meta-analysis [file sj-docx-4-tag-10.1177_17562848251378236.docx]

**Appendices 2-5**

**Appendix 2a: Summary of Jadad score for music RCTs**

| **Study** | **Randomisation (2)** | **Blinding (2)** | **An account of all patients (1)** | **Total (5)** |
| --- | --- | --- | --- | --- |
| **Celebi et al 2020^31^** | 1 | 0 | 1 | 2 |
| **Costa et al 2010^32^** | 2 | 2 | 1 | 5 |
| **De Silva et al 2016^20^** | 1 | 2 | 1 | 4 |
| **Martindale et al 2014^33^** | 1 | 0 | 1 | 2 |
| **Ovayolu et al 2006^34^** | 2 | 0 | 1 | 3 |
| **Bechtold et al 2006^35^** | 2 | 1 | 1 | 4 |
| **Lee et al 2002^36^** | 2 | 2 | 1 | 5 |
| **Brix et al 2022^37^** | 2 | 0 | 1 | 3 |
| **Binek et al 2003^38^** | 0 | 0 | 1 | 1 |
| **Cakir et al 2023^15^** | 2 | 0 | 1 | 3 |
| **Ko et al 2017^39^** | 1 | 0 | 1 | 2 |

**Appendix 2b: Summary of Jadad score for distraction RCTs**

| **Study** | **Randomisation (2)** | **Blinding (2)** | **An account of all patients (1)** | **Total (5)** |
| --- | --- | --- | --- | --- |
| **De Silva et al 2016^20^** | 2 | 1 | 1 | 4 |
| **Han et al 2021^16^** | 2 | 0 | 1 | 3 |
| **Calkir et al 2021** | 2 | 0 | 1 | 3 |
| **Liu et al 2022^28^** | 2 | 1 | 1 | 4 |
| **Cakir et al 2023^15^** | 2 | 0 | 1 | 3 |
| **Umezawa et al 2015^29^** | 2 | 2 | 1 | 5 |
| **Xiaolian et al 2015^17^** | 2 | 0 | 1 | 3 |
| **Lee et al 2004^18^** | 2 | 0 | 1 | 3 |
| **Sheng et al 2020^19^** | 2 | 0 | 1 | 3 |
| **Shamali et al 2024** | 2 | 0 | 1 | 3 |
| **Veldhuijzen et al 2020** | 2 | 0 | 1 | 3 |
| **Yilmaz et al 2021** | 2 | 0 | 1 | 3 |

**Appendix 3: Music RCT pain score % change**

**Appendix 4: Music RCT anxiety score % change**

**Appendix 5: Publication bias statistical analysis**
